# Application of Internet of Things and Sensors in Healthcare

**DOI:** 10.3390/s22155738

**Published:** 2022-07-31

**Authors:** Mohammad S. Al-kahtani, Faheem Khan, Whangbo Taekeun

**Affiliations:** 1 Department of Computer Engineering, Prince Sattam Bin Abdul Aziz University, Al-Kharj 16273, Saudi Arabia; alkahtani@psau.edu.sa; 2 Department of Computer Engineering, Gachon University, Seongnam 13120, Korea

**Keywords:** COVID-19, healthcare system, Internet of Things, IoT application, sensors, smart technology

## Abstract

The Internet of Things (IoT) is an innovative technology with billions of sensors in various IoT applications. Important elements used in the IoT are sensors that collect data for desired analyses. The IoT and sensors are very important in smart cities, smart agriculture, smart education, healthcare systems, and other applications. The healthcare system uses the IoT to meet global health challenges, and the newest example is COVID-19. Demand has increased during COVID-19 for healthcare to reach patients remotely and digitally at their homes. The IoT properly monitors patients using an interconnected network to overcome the issues of healthcare services. The aim of this paper is to discuss different applications, technologies, and challenges related to the healthcare system. Different databases were searched using keywords in Google Scholar, Elsevier, PubMed, ACM, ResearchGate, Scopus, Springer, etc. This paper discusses, highlights, and identifies the applications of IoT healthcare systems to provide research directions to healthcare, academia, and researchers to overcome healthcare system challenges. Hence, the IoT can be beneficial by providing better treatments using the healthcare system efficiently. In this paper, the integration of the IoT with smart technologies not only improves computation, but will also allow the IoT to be pervasive, profitable, and available anytime and anywhere. Finally, some future directions and challenges are discussed, along with useful suggestions that can assist the IoT healthcare system during COVID-19 and in a severe pandemic.

## 1. Introduction

### 1.1. Overview

Today we are living in a digital world where things/devices are interrelated with one another. The internet makes them smart devices when these devices are connected with one another through the internet. The Internet of Things (IoTs) is a large network of interconnected devices that store and collect data about their nearby environment, and therefore the IoT is considered as an ecosystem of the interconnected devices. The IoT is very important in accessing and regulating digital devices remotely within the current structure. This produces an opportunity for digital devices and physical items to enhance community, methodology, and commerce. The devices vary from a nanochip to a router and are utilized along with sensors, actuators, and software to communicate with one another. The IoT has wide-ranging applications and is growing very quickly. 

Almost all the monitoring applications in this digital world totally depend on wireless sensor networks (WSNs) due to their undeniable advantages, such as lower cost, less infrastructure, different network topologies, less maintenance, etc. Wireless sensors and WSN are providing monitoring services in almost every aspect of life, such as climate change, weather, natural disasters, traffic, forests, healthcare, location, etc. In healthcare services, the important objectives of the sensors are to regulate, monitor, control, warn, and track the activities of the patient. Sensors analyze and diagnose such activities in a hassle-free manner and make the healthcare system self-dependent and free from physical intervention. As a result, healthcare will speed up in real time and exponentially increase. 

**Figure 1:**
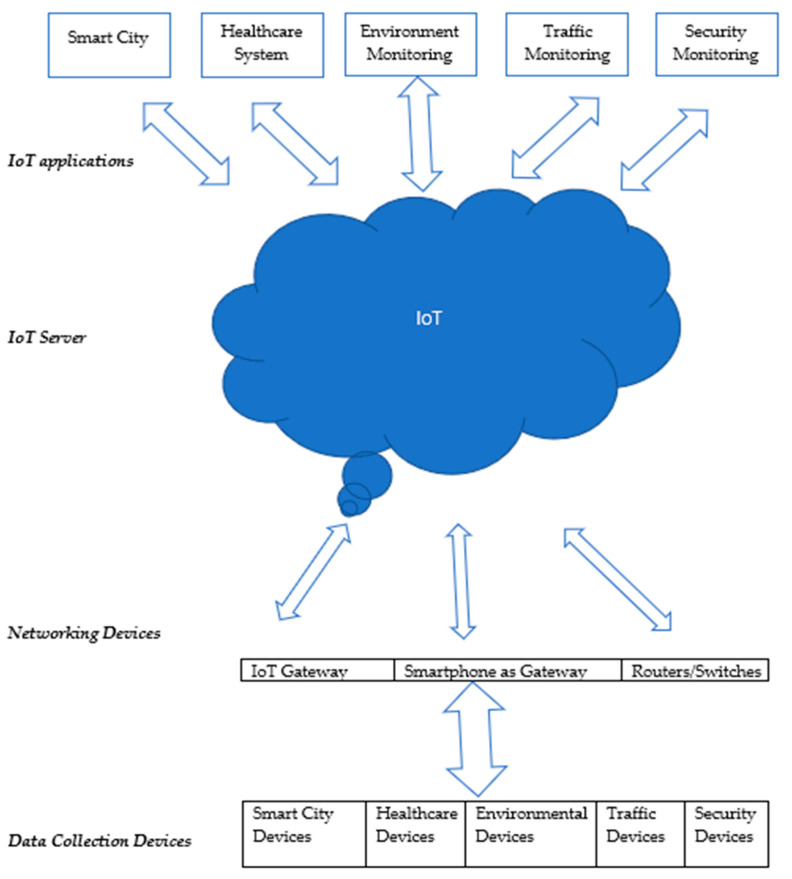
IoT network.

Previously, numerous healthcare applications [1,2,3,4,5] relevant to the IoT have been proposed to facilitate patients, doctors, and administration. These applications are used for the betterment of the existing healthcare system by contributing real-time intensive care about the patient’s illness, medical emergency management, etc. IoT applications allow the patient to watch and record existing health disorders, such as blood pressure, insulin level, heart disorder, physical suitability, etc., as data and forward them to a designated health center or physician. Healthcare midpoints or clinics are also utilizing IoT applications extensively, and they deliver quick facilities by discovering their position and health disorder, as shown in Figure 1.

**Figure 2:**
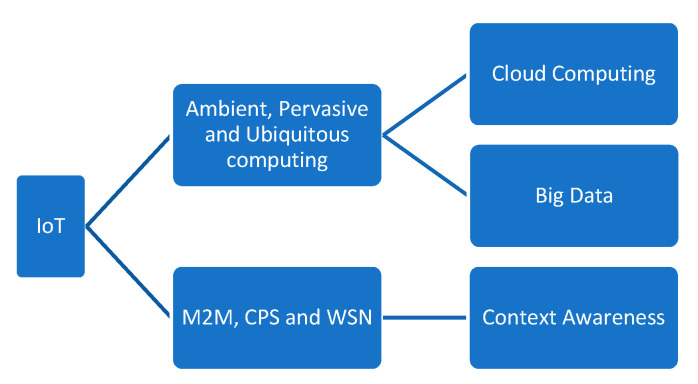
Relationship of the IoT and related technologies.

In Figure 2, ambient, pervasive, and ubiquitous computing form the basis of the IoT. The relationship between machine-2-machine (M2M) communication, cyber-physical systems (CPS), and wireless sensor networks (WSN) with the IoT is discussed. M2M and WSN technologies form the basis of the IoT. To develop interactive applications on M2M, CPS provides robust coordination between physical objects and computational elements. M2M, CPS, and WSN technologies have ability in actuation, sensing, and computation. The IoT is a robust WSN that depends on M2M and provides the ability to produce CPS. 

Cloud technologies offer easy backup and storage, processing, analysis, and management of large amounts of data to the IoT with lower cost with enhancement in fault tolerance. Traditional data processing is not useful in this modern technology and, hence, the IoT depends on big data technologies for analyzing and processing. Similarly, the IoT with process information is merging with context aware technologies to assist in searching, filtering, and interpreting huge amounts of sensor data. 

### 1.2. Background

The research community and industry are exploiting IoT applications with real-time integrated devices to improve the life of the common user. The IoT is getting more use at every institution, particularly in the healthcare system by offering clinical facilities, nursing for patients, innovative searching and monitoring of medical difficulties, computer-based therapy, and consistent backup facilities for patients, especially during COVID-19 [6]. The smartphone accelerates these processes by determining and gathering patient information continuously [7]. The regular information collection and monitoring are achievable through sensors that are applied and mounted in the wards, tools, and emergency facilities to continuously transfer the data to the adjacent healthcare organization [8] for rapid response. Through the IoT, healthcare units are contributing a well-organized and consistent tracking system to society [9]. Similarly, cloud computing is also contributing through data storage, distribution of facilities, combination of data facilities, and primary safety warnings to the healthcare system [10].

Some studies have briefly considered integrating the healthcare system with the IoT. For example, currently almost all civilians are aware of the symptoms of the coronavirus. Hence, a group of well-informed, interconnected people in a society may be selected to report infected people to the concerned authorities. This will minimize the risk of spreading the virus in society. Similarly, some smartphone-based applications could be introduced in the community, so people could get a fast emergency response with a medical facility [11,12,13,14]. In the current typical situation, there is no protocol defined for lockdown. Worse problems arise due to non-accessibility of the resources to the patient. Hence, through the IoT, it will be possible to access the patient in a remote location [15,16]. Paper [17] covers communication technologies, such as wired networks, mobile ad-hoc networks, wireless networks, and several elements of the sensor network [18,19], that are necessary for the IoT. In [20], IoT architecture is discussed in detail, with challenges related to the development and deployment of IoT applications in a healthcare system. The centralized cloud vision and the enabling technologies of IoT applications are discussed in [21]. Survey [22] presents the IoT for specific wireless devices using 802.15.4, near field communication (NFC), 6LoWPAN/IEEE, Bluetooth for mHealth, and eHealth applications. Papers [23,24] show the uses and challenges of the IoT with radio frequency identification (RFID) and optical wavelength [25], along with its potential applications.

### 1.3. Contribution and Scope

This paper explains the up-to-date procedures relevant to IoT healthcare-based systems using sensors. We note the current research in IoT healthcare systems, but the main emphasis of this paper is to assist the healthcare system from the IoT. Furthermore, this paper offers new guidelines to the academic/research community in user-friendly IoT applications.

**Figure 3:**
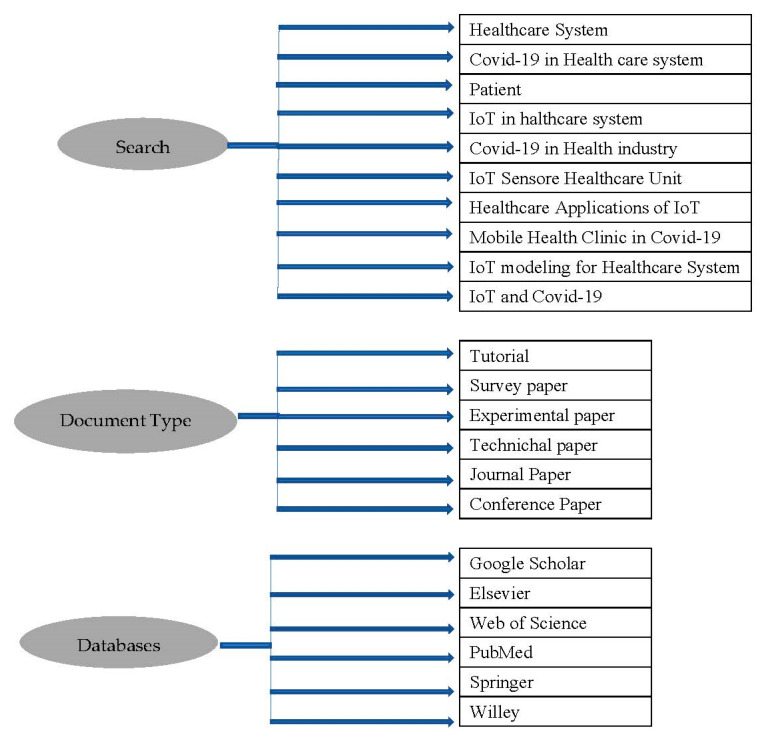
Data collection criteria.

To recognize the services of the IoT, it is necessary to recognize the architecture and elements of the IoT. Consequently, a review is shown in Figure 3, and only those IoT healthcare applications that could enable the healthcare system to offer more facilities to the patient are discussed in this paper.

We discuss many significant IoT applications using sensors to maximize patient care, improve the workflow, increase the effective operation of limited resources, reduce cost to the patient, etc. Many technologies are highlighted and recommendations are given about utilizing several techniques in different circumstances, patients, illnesses, positions, etc.

Finally, many challenges and impacts for the healthcare system, especially during COVID-19, are highlighted. These challenges include security and confidentiality issues, procedural issues, technical issues, economic issues, etc.

This paper expansively emphasized the applications, technologies, challenges, and future directions related to healthcare systems. This paper is a state-of-the-art survey that not only benefits society and the healthcare system but may also be very valuable for a more serious future pandemic.

### 1.4. Organization of the Paper

**Figure 4:**
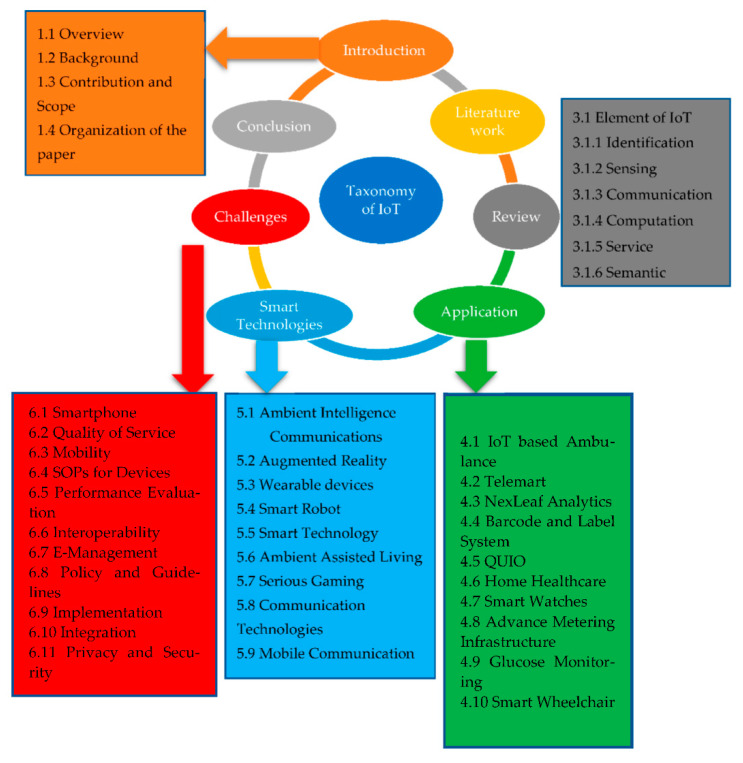
Taxonomy.

This paper is divided into seven sections, as shown in Figure 4. 

Section 1 describes the introduction, overview, and background of the IoT healthcare system. Contributions are described with explicit evidence. Section 2 explains the related work relevant to IoT healthcare systems. Section 3 describes the IoT elements to better understand the healthcare system. Section 4 discusses almost all the relevant applications related to the IoT healthcare system. The healthcare system practices all these applications that are used during COVID-19 and for upcoming pandemics to assist society against any pandemics. Section 5 explains the technologies in detail. Section 6 discusses the future direction and challenges relevant to the IoT healthcare system and provides more information than previous papers. Finally, Section 7 concludes the paper.

## 2. Literature Work

This article reviews the IoT and healthcare systems in parallel. In the first phase, several papers were collected relevant to the healthcare system. In the second phase, the articles relevant to the IoT healthcare system and sensors were collected. IoT and sensors keywords relevant to healthcare systems were searched. Different databases were examined with the search terms, as shown in Figure 3. 

The number of diseases and patients are quickly increasing around the globe and making the healthcare system complicated. It is necessary to design a well-ordered and systematic IoT methodology. The IoT is now facilitating numerous organizations and industries to fulfil objectives, such as the Internet of Healthcare Things (IoHT) and the Internet of Medical Things (IoMT). Using the procedures and services presented by the IoHT and the IoMT, the problems of healthcare systems can be controlled and minimized for almost all kinds of diseases, including COVID-19.

In [26,27], the IoT and cloud computing are used in parallel with the healthcare system. IoT applications are proposed to tackle many challenges, and they use a cloud-based model to provide services to identify a patient from the home. A healthcare system is executed through Android applications to cloud IoT and computing. In [28,29], a new approach for the healthcare system was designed due to the demand in the IoT within academia, industry, and society. These two articles describe a system for monitoring heart rate through a smart health band, and the patient’s information is communicated to his friends and family members. In [29], the heart patient is informed through an initial discovery system known as iCarMa. In iCarMa, the patient’s condition is detected and diagnosed early and then communication is performed. Currently, the IoT has developed the important part of the medical aid system, and patient statistics should be kept secure from unlawful users. 

Survey [30] was specifically designed for privacy and security within the context of the IoT. The authors discuss that the cloud in the IoT introduced a risk factor in the medical care system. In this paper, suggestions were made to motivate the researcher to resolve the issues related to the privacy and security of medical care systems within the context of the IoT.

Paper [31] proposes the integration of the IoT with cloud computing in the healthcare system. Many challenges are highlighted within the context of the IoT that affect the healthcare system and should be resolved to improve it. In this paper, the audio pathology model is proposed for monitoring the patient. The main challenges in this model are the ease of use and interoperability of the framework. It is suggested that the integration of different voice models can accomplish the scalability of the dynamic nature.

Paper [32] discusses various applications, industrial importance, technologies, and the framework of the IoT. It discusses the security issues related to the needs of security, features of security, the privacy of the IoT, and model requirements to avoid taxonomy attacks. An intelligent model was proposed to reduce attacks and introduce further IoT enhancements related to the medical care system and e-health. Finally, the paper highlights the importance of the IoT in the medical care system by describing guidelines for future researchers, policymakers, and engineers in the IoT. Paper [33] discusses the prediction of early symptoms through the IoT, which are useful for the patient and a very beneficial tool for the society. It discusses all the papers related to reviews and challenges associated with the IoT in the healthcare system. At the end of the paper, some suggestions are proposed to improve the healthcare system through different IoT techniques.

Article [34] explains that communication systems improve the practices of the IoT in organizations and industries, particularly in healthcare systems. This article proposed a body sensor network that can communicate data efficiently and constantly to a public IoT-based system. It also proposed some necessary security constraints that could improve the security of the planned procedure and offered suggestions for the future system on portable objects.

All the papers noted above are valuable for detecting patient disorders and then communicating the data to the healthcare system in order to assist the patient. In this current article, all patient services through the IoT are discussed and recommendations are proposed for the healthcare system for addressing the risks for diabetic and heart patients. This article proposes novel IoT-based technologies and healthcare applications during acute situations, where the patients are worsening with the passage of time. The IoT is still a new technique for academia and healthcare professionals, but its use is unavoidable in the healthcare industry due to its economic cost and real-world solutions.

## 3. IoT Overview 

### 3.1. Elements of the Internet of Things

**Figure 5:**
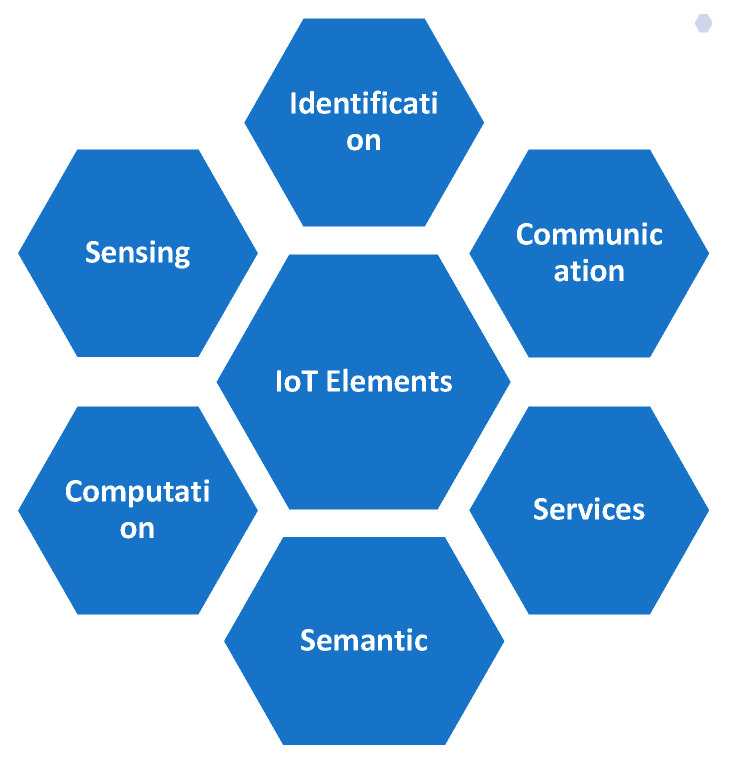
Elements of the IoT.

To understand the function and services of the IoT, it is very necessary to understand each and every element. There are six main elements of the IoT along with categorization and examples, as shown in Figure 5, which help the IoT to function properly.

#### 3.1.1. Identification

It is very important that the IoT recognizes the customer from its name and address whenever the user demands and that it will clearly recognize each object within the network. There are many identification methods offered for the Internet of Things, such as ubiquitous codes and electronic product codes. In identification, addressing is very important in the IoT, because addressing helps to differentiate between an object ID and an address. Object ID could be any physical object in the IoT, such as a sensor, actuator etc., and object address refers to its address with the communication range of the network, such as IPV4 and IPV6. IPV6 is very useful in the IoT due to the implementation of low-power wireless networks. Furthermore, objects in the communication network can use public IP and not private IP.

#### 3.1.2. Sensing

Sensing is the gathering of information from all devices within the predefined network and then transmitting that information to any data warehouse or cloud for further processing. The collected information is due to some specific tasks based on some specific services. To accomplish more benefits from the IoT, smart actuators and sensing devices are required. Many industries, such as WeMo and SmartThings, are providing smart devices and applications to maintain, update, control, and monitor hundreds of smart devices inside a campus or building by using smartphones [35,36,37]. Microcontrollers, computers, sensors, built-in TCP/IP, and security measures are usually used to understand the importance of IoT products. All devices are connected to a central management service to offer the data required by the customer. 

**Figure 6:**
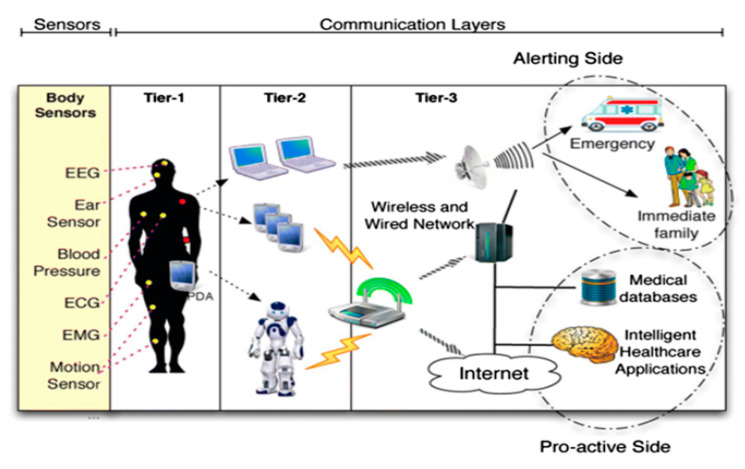
Sensors using the IoT in healthcare systems.

Sensors are the backbone of any application calculating and handling the proposed data for sensor variations in the physical world, as shown in Figure 6. Several sensors are introduced in the market, varying from simple to complex. The organization of sensors is totally based on the requirement, specification, method of conversion, experiment and material type, sensing methodology, measurements properties, and field of applications [38,39].

#### 3.1.3. Communication

In the IoT, the communication between heterogenous objects is possible through different communication protocol, such as near field communication (NFC), Wi-Fi, radio frequency identification (RFID), ultra-wide bandwidth (UWB), Bluetooth, IEEE 802.15.4, long term evolution (LTE), Z-wave, etc.

NFC usually operates at a higher frequency band of 13.56 MHz with a data rate up to 424 kbps. The communication between two active readers and passive tags is up to 10 cm [40]. Wi-Fi is a technology that is used for data communication within a 100 m range, and it can be used efficiently for data communication between heterogenous objects without using a router in a mobile ad-hoc environment [41]. RFID first recognized the concept of machine to machine (M2M) in the field of communication technology, and it is the exchange of communication between the machine or some other devices in industry and private infrastructure. RFID is composed of an RFID reader and RFID tags. The RFID reader detects the RFID tags and receives the information from the reflected signal of the tag. The information is then transferred to a database within a radio range from 10 cm to 100 m [42]. UWB technology uses low energy and high bandwidth. The application of UWB related to sensors is increasing because it is favorable in a situation where the communication occurs in a low coverage area [43]. Bluetooth is a short-wavelength communication technology for data communication between devices over a short distance. It is very useful in the IoT because of the lower energy consumption and high-speed connectivity over a short distance [44].

LTE is a standard for wireless communication for high-speed data on a mobile phone [45]. It uses the services of broadcasting and multicasting and can be useful in a highly mobile situation where frequent topology changes occur, up to 100 MHz, with uplink and downlink spatial multiplexing extended in the advanced version of LTE bandwidth support [46].

#### 3.1.4. Computation

Processing units (microprocessor, microcontroller, field-programmable gateway array, system on a chip), hardware platforms (Raspberry Pi, Mulle, T-Mote Sky, Arduino, intel Galilio, UDOO, etc.), and software platforms shows the computation capacity of the IoT. The operating system is the most important among the software platforms and runs during the activation time of the system. Real-time operating system (RTOS) and Contiki RTOS are widely used for the development of IoT-based applications. The cloud provides a wide platform, and it is a key part of the IoT. The role of the platform is to facilitate the smart devices to transmit the information to the cloud for real-time processing through big data. As a result, the user receives the information that is extracted from the big data.

#### 3.1.5. Services

The IoT services are categorized into identity-related, ubiquitous, collaborative-aware, and information aggregation services. Identity-related services are the basic services that are very useful in the functionalities of other services. All the applications that want to show real world objects in the virtual world have to identify such objects. The information aggregation services gather, process, and report the collected sensor information to IoT applications. Collaborative-aware services make decisions and react accordingly on the information of aggregation-related services. Ubiquitous services are the service provider to the collaborative aware services and hence can provide services to anyone and at any location [47,48,49]. 

A review of different IoT applications is very simple under the background of the aforementioned services. The majority of IoT applications consist of collaborative-aware and information aggregation services. Smart campus/building, intelligent transport systems, smart industry, and smart home belong to collaborative-aware services. Smart healthcare systems, smart education, and smart grids belong to information aggregation services. Similarly, smart city application belongs to ubiquitous services, and they improve the quality of life by providing services like transport, utilities, and health, etc.

#### 3.1.6. Semantic

This is the ability to extract information from different devices in the IoT and then offer the required services to the user. Extraction contains discovering information, uses of information, and modeling information. It contains analysis about decisions and use of the correct service of data extraction and then fulfills the correct demand to the correct customer/user [50]. This demand of the resources is provided by the web ontology language (OWL) and resource description framework (RDF).

## 4. Applications and Gadgets for the IoT Healthcare System

The IoT and sensors are the most important parts for from a technology perspective because they facilitate the healthcare system and hence are directly related to the user or patient requirements. Currently, multiple applications are used that are user-centric, whereas services are developer-centric because services are used for developing applications. Hence, the integration of technology has allowed the IoT to be the most efficient and reliable technology for the healthcare system as well as for COVID-19 [51]. Various applications for the healthcare system are discussed in this section.

### 4.1. IoT Based Ambulances

The job of ambulance staff is very stressful because they deal with more serious patients, and a timely decision is critical concerning the patient’s life. However, an IoT-enabled ambulance is very effective due to a remote medical team suggesting necessary action related to the patient. Hence, the patient is responded to in a timely manner with effective measures in IoT-enabled ambulances [52]. Red Ninja was the first company to develop a Life First Emergency Traffic Control (LiFE) algorithm that changes the traffic light pattern or duration during an emergency for ambulances and emergency service providers [53,54].

### 4.2. Telemart

Telemart is helpful in the IoT because it is challenging to maintain a 6-ft distance in a supermarket, for example, during COVID-19. Recently, Amazon installed Telemart to provide a QR code for the store entrance to the users, who are then allowed to go shopping. After shopping, the money is debited from the user’s Amazon account, and the customer collects all the purchased items. Thus, the customer is able to pay without waiting in line for the counter [55,56] and, therefore, without compromising social distancing requirements.

### 4.3. Nexleaf Analytics

This application is very useful in developing countries for both cooking and vaccines. With the help of this application, the temperature of a life-saving vaccine in a refrigerator is continuously monitored [57]. These vaccines are placed in the healthcare systems/clinics in remote or rural areas. is the application is also helping to improve cookstoves, which send real-time data about the emission of carbon dioxide and wood fuel. Hence, this system measures COVID-19 patient data from a remote location because it has the system and infrastructure for measuring and transmitting data to the cloud and the server.

### 4.4. Barcode and Label System

This system is a wireless cloud platform that interconnects multiple therapeutic devices for the treatment and health monitoring of patients with chronic disease. It also allows mobile and web-to-healthcare units and medical teams to respond quickly by using the real-time data of the patient [57,58,59]. Furthermore, it developed a platform of drug delivery by using hardware and software to increase the link between the user and the system for the detection of the diseases. The concept of virtual hospitals and virtual wards is used almost everywhere in the UK, Australia, America, and the Middle East. The concept of a virtual hospital was implemented in Sydney, Australia, in 2020 during the COVID-19 pandemic [60]. The virtual hospital offers a remote service to the patient with the coronavirus using measuring and monitoring of pulse rate, heart rate, and temperature. The data are uploaded on the server and then communicated to the patient and virtual hospital through a mobile application.

### 4.5. Quio

According to the WHO, the expected lifetime of people will increase beyond the age of 60 due to everyday facilities and availability of medicine and medical care worldwide [61]. The number of aging people will outnumber the beds in hospitals, and soon healthcare services will need to be provided to them at their homes. Hence, it is expected that in 2030, their homes will be converted into their hospitals, and all the hospital care services will be transformed into home care services [62]. The home-based service, connected through the IoT, is a promising solution in the COVID-19 situation. For example, Parkinson house is a combined project of IBM and Pfizer that uses IoT technology. This increases the doctor–patient relationship by monitoring drugs’ effectiveness and adjusting medications if required by the patient in real time. The house is fully equipped with a sensor to monitor every patient’s movement and then send the data wirelessly to the concerned physician. The doctor checks the patient’s progress and response to the medication through the data received from the house.

### 4.6. Home Healthcare

Initially, smartwatches [63] were only for entertainment and other activities. However, because of the multiple applications from the manufacturers such as Samsung, Apple, and Google, it is the most powerful healthcare tool currently. For example, the Apple watch series 4 introduced the echocardiogram (ECG) to detect atrial fibrillation (AFib), a heart condition that can cause a blood clot, stroke, etc. In the Apple watch series 6, a feature to monitor respiratory diseases, such as asthma, is also included. Similarly, Fitbit and Samsung Galaxy also introduced the ECG and blood pressure monitors in their watches for users. Other health-related applications are also included in the watches, such as running, steps count, sleep cycle, tracking, movement reminders, medication reminders, etc. New applications are coming in the new watches of Apple, Google, and Samsung, and now COVID-19 related reminders are also added. Hence, people regularly wash their hands with soap and properly sanitize their hands.

### 4.7. Smart Watches

This application is designed for aged people who live alone with no relatives. This app is very useful for serious health issues, accidents or falling, or taking medication when they cannot get any support or help. In this app, the patient uses a wearable pendant that detects a fall, sudden movement, or no movement for an abnormal amount of time after sudden changes [64]. As a result, a message is communicated to the emergency services with the patient location. This app was very useful during the COVID-19, which prohibited many family members from visiting aged relatives and protected them from the coronavirus.

### 4.8. Advanced Metering Infrastructure (AMI)

During pandemic crises, the smart grids need to provide uninterrupted power supply to the hospitals, healthcare units, and any healthcare infrastructure. Smart grids consist of sensors and transducers used to monitor the supply and demand of electricity [65], and the AMI performs this task. The AMI is installed by the electricity providers to remotely detect, control, and prevent technical issues. AMI in a smart grid has many smart meters, and these smart meters are connected with the internet through the IoT. As soon as a meter reading is collected, it is communicated to the main sever of the electricity providers. During COVID-19, this application was essential for the safety of both the workers and the customers by limiting interaction. Through AMI, remote data collection of the meters and system refreshing is possible during a fault. Through AMI, it is also possible to locate the dangerous zones declared by the government during COVID-19 and to provide uninterrupted power supply to that specific location or health infrastructure.

### 4.9. Glucose Monitoring

Devices for monitoring glucose level, blood pressure, and temperature are ubiquitous in society, and they have played an essential role in COVID-19. Diabetic patients may have multiple metabolic diseases, such as high sugar level for an extended period. Monitoring sugar level shows the pattern of blood glucose variations and assists with daily activities such as eating, drinking, and taking medication. A technique is proposed [66] for sensing glucose level in real time, and the data are uploaded to a server and then sent to specific physicians for further processing. The sensors are connected without interruption with the IPV6 for data exchange to the concerned healthcare service provider. This exchange of data between the patient and the service provider is possible because of the IoT. The same procedure is now used to detect and prevent COVID-19 pandemics.

### 4.10. Smart Wheelchair

Much research has been conducted to develop smart electric wheelchairs for disabled, injured, and older people. Smart wheelchairs can go to the market, the healthcare center, and especially to the hospital during the pandemic. This smart wheelchair is connected wirelessly with many sensors that are interconnected with the IoT [67,68]. The patient’s data are detected from the movement of the wheelchair, the surrounding environment, the location of the user, etc.

## 5. Smart Technologies of IoT Based Healthcare Systems for COVID-19

Currently, many technologies from the IoT are used by healthcare systems. The use of IoT technologies is increasing very rapidly due to COVID-19. Many new technologies from the IoT are introduced over time according to the demand of the user and industry. Some of the technologies that are and can be very useful for future IoT healthcare systems are as follows:

### 5.1. Ambient Intelligence Communications Technologies

The application of ambient intelligence is significant for the users and patients. It is finding an application in the healthcare system by assisting patients, physicians, and staff of the healthcare center [69]. It includes wearable devices and sensors integrated with healthcare settings and the IoT to collect and interpret patient data. Hence, the embedded system of human–computer interaction, autonomous control, the IoT, and ambient intelligence during COVID-19 can assist the government, healthcare systems, and patients.

### 5.2. Augmented Reality (AR)

In the modern era of the Industry 4.0, augmented reality is an essential pillar in the advancement, learning, and creation of new technology. The introduction of information technology (IT) into the healthcare system is very revolutionary. For example, AR is used to guide medical experts in medical training and education, remote monitoring, and surgery. Similarly, AR encourages and educates the community about handwashing to prevent COVID-19 and other diseases [70].

### 5.3. Wearable Devices

**Figure 7:**
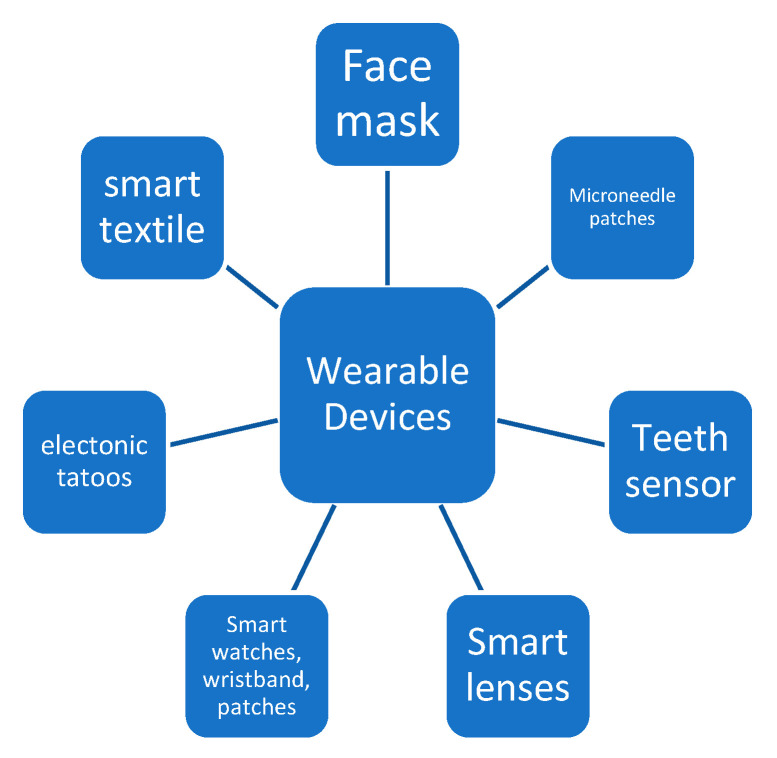
Wearable devices.

Wearable devices assist the patient and the healthcare system with many health issues at a low cost. These devices integrate with many sensors and wearable accessories, such as smartwatches, smartphones, necklaces, wrist bands, etc., and collect data about the patient and the surrounding environment. The information is uploaded to servers and accessed through a smartphone [71,72]. The interconnection of mobile application with IoT wearable devices increases the computing of the devices. Wearable devices, as shown in Figure 7, are now a commonplace part of the healthcare system using the IoT, and they are very useful in combating pandemics such as COVID-19.

### 5.4. Smart Robots

**Figure 8:**
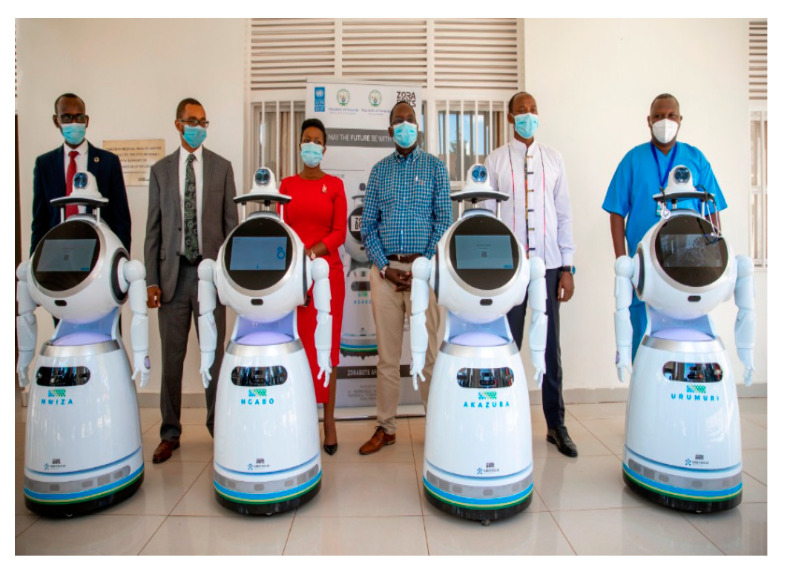
Smart robots.

Robots are playing an important role, as shown in Figure 8, against COVID-19 by the UNDP in Rwanda to make life easier and smarter than ever before [73]. During COVID-19, the IoT and robots are combined to help the industry and government against the COVID-19 pandemic. Robots, with the help of the IoT, are used to drop the infectious items of the patient into the trash, move objects of the patient from one place to another place around the hospital, clean the room and hospitals, disinfect the rooms, etc. This robotic technology, along with the IoT, introduces a new working environment to the world. Germ-zapping robots [74] use ultraviolet light to do the automatic cleaning all across hospitals and have the ability to continue proactively cleaning around the clock. This technology is now used in other sectors, such as train stations, universities, grocery stores, movie theatres, shopping malls, homes, etc.

### 5.5. Smart Technology

COVID-19 has led the industry to redefine their working procedures and also to change their working environment. According to an MIT review, some examples, are spacing out desks, removing conference room chairs, installing antimicrobial surfaces, adding thermal scanners, altering air-conditioning systems, applying floor markings, and mandating rigorous cleaning protocols. Some are going further still: offering or even requiring coronavirus testing for workers returning to the office. The most advanced industries were not prepared for a situation such as COVID-19. These industries are shifting towards the automation of their smart devices and robotic tools to ensure their employee safety and increase their work efficiency by deploying a “Command Center” [75], which converts all the innovative technology into business intelligence technology. The command center collects all the industry data for decision-making about strategy and execution in critical times and situations.

### 5.6. Ambient Assisted Living (AAL)

To improve health, safety, and quality of life and to preserve honor, an intelligent system of assistance, i.e., AAL, is introduced for older people in society. This facilitates the support staff, doctors and the healthcare system during the care for older people [76,77]. For COVID-19, it is combined with wearable devices, smartphone technology, and the IoT. It allows caregivers, family members, and friends to inform older people about any emergency and the hazardous situation in the house or healthcare center.

### 5.7. Serious Gaming

Serious gaming is getting much attention during the lockdown for COVID-19, and it has been designed for some important purposes other than pure entertainment. Serious gaming is used for education, healthcare, scientific research, training, engineering, and many other fields. It can be used to facilitate learning and training in the areas noted previously. During COVID-19, the patient’s condition, exercise, and other activities can be carried through serious gaming, which is very useful for COVID-19 patients [78,79].

### 5.8. Communication Technologies Related to Healthcare

The technologies of healthcare related to the IoT are divided into identification, communication, and location technology. In identification, a unique identifier (UID) is assigned to each sensor or node in the healthcare system to easily access patient data. In addition, the patient in a remote location can easily use the healthcare system’s resources through UID. UID is further divided into universal UID and global UID. The former is a part of distributed computing and operates in a decentralized administration. There is the possibility that its components change in universal UID due to regular upgrades of IoT technologies, and hence global UID is required. This will help make its global directory search for IoT services under the UUID scheme [80]. 

Communication technology assures the connectivity between different devices in the healthcare network. Communication technology is further classified into short, medium, and long-range communication. Short-range communications are used for a limited area, especially for body area networks (BANs). Medium range communication is used to interconnect devices at a large distance, such as communication through Wi-Fi between a server and central node of the BAN. Long-range communication is used for a more considerable distances compared to medium range communication, such as satellite communication. The most common technologies for users are RFID, NFC, Bluetooth, Wi-Fi, Zigbee, satellite communication, worldwide interoperability for microwave access (WiMAX), etc. In location-based technology, a real-time location system is used for the treatment of the patient with the resources available at patient’s position within the healthcare system. The global positioning system (GPS) is a widely used technology for tracking purposes. All communication technologies are used to locate a COVID-19 patient, ambulance, emergency services, healthcare centers, vaccination centers, etc.

### 5.9. Mobile Communication

Mobile communication has gone through many changes from the first generation to the sixth generation. The first generation used an analog system for real-time communication, and the 2G used a digital system. The online exchange of information was possible by the introduction of 3G. To overcome the limitation of 3G, 4G was introduced, increasing the quality and services and decreasing the cost of the resources. Similarly, 5G [81] is offering more capacity, bandwidth, and speed compared to 4G. Currently, work is in progress on 6G to unify satellites for more coverage within a larger covered area [82,83].

## 6. IoT Based Healthcare Challenges/Limitations for COVID-19 and Future Pandemics

The demands on the IoT are increasing in different industries, especially in the IoT healthcare system, and much research is done on designing and executing IoT-based healthcare applications. However, multiple matters in IoT-based applications are very important and require special consideration to improve applications and services. Many databases, including IEEE, ACM, Google Scholar, Willey, Elsevier, Springer, Scopus, etc., were examined for the new challenges in the IoT healthcare system. To overcome these challenges, the IoT must lessen the distance between healthcare system and patient during COVID-19 and future pandemics.

### 6.1. Smartphone

A smartphone plays a vital role along with the IoT in healthcare systems, and the patient and the physician can check the data in real-time. The IoT introduces many applications for the healthcare system. However, some specific types of applications are necessary that show early detection of symptoms of disease or the user’s visit to an infected area. For example, a patient having symptoms of COVID-19 or the patient’s presence in an infected area should be reported to the healthcare system for further actions.

### 6.2. Quality of Service (QoS)

Quality of service in terms of reliability, efficiency, and service level is essential in healthcare systems. Here, reliability is defined in term of digital services and efficiency in term of timely response, and both parameters are very useful for the IoT framework. The quality of service, especially in a healthcare system, should not be compromised, because negligence can put a life in danger. The service provider should provide an alternate method if the system fails. 

### 6.3. Mobility

IoT healthcare-based systems should support a patient’s mobility from one place to another or during a move. For example, in some areas, a wired network is not available, so the patient should be connected with a wireless network or a mobile network. However, if none of the services is available, he should be connected through the mobile ad-hoc network (MANET) [84,85,86,87].

### 6.4. Standard Operating Procedures (SOPS) for Devices

Thousands of sensors and other devices are required to fully deploy the IoT in healthcare systems. These devices are made of semiconductors and can cause more complications if implemented on the human body. Therefore, care must be considered, and guidelines are required during device manufacturing. In addition, the radio spectrum should be within the safe zone for data communication because too many devices and radiofrequencies can negatively impact human health, animals, and even the entire ecosystem. Therefore, guidelines should be followed properly during the manufacturing and implementation of the devices. 

### 6.5. Performance Evaluation

To evaluate the performance of IoT services, it is compulsory to assess the performance of the underlying technologies and the performance of the components to increase user satisfaction and meet the desired requirements of the industry. Many parameters are used in the industry for performance, such as processing speed, cost, packet loss, response time, availability of the services, speed of the communication services, delay, throughput, etc. Many open issues of IoT applications need to be resolved.

### 6.6. Interoperability

To manage and operate different heterogeneous devices from other platforms is a very challenging task. However, regardless of the hardware platform, there should be a consensus between the application developer and IoT designer to guarantee the availability of services to all healthcare system users. The most common examples are mobile communication technologies like GSM and Wi-Fi that ensure interoperability by using different platforms.

### 6.7. E-Management

In the coming years, this area will face most of the challenges due to the transition of the traditional healthcare system to an IoT-based healthcare system. Furthermore, millions of sensors and devices in healthcare will be introduced in the future due to the COVID-19 pandemic. Therefore, it will be the responsibility of the service providers to manage technical error, configurations, reliability, security, and performance to facilitate the users and the healthcare system. Management should increase their efforts to develop lightweight protocols, devices, and applications to provide better services.

### 6.8. Policy and Guidelines

The IoT is the most developing industry in many countries, and guidelines and policies are in the initial stages for the privacy and data of the user. The Australian Privacy Principle (APP) developed its data privacy and user protection guidelines in the IoT. Similarly, the U.S. Dept. of Transportation (USDOT) is financing $160 million in the next five years in smart transportation, smart universities and cities, healthcare system etc. [88]. Therefore, they are preparing different guidelines and policies for that area to protect the privacy and data of the user, especially in COVID-19.

### 6.9. Implementation

Smart communities need millions of low-cost, low-power sensors and devices; it is not easy to maintain, implement, and update such an extensive and intelligent infrastructure. Fault tolerance and resistance are the two most important challenges for reliable data delivery between sensors and embedded devices. Another critical challenge is the cost of the devices used in communication, operation, and infrastructure. Therefore, there should be some tradeoff between the cost and the IoT application. This tradeoff is essential during COVID-19 because the user paid more than the actual rate of the application or facility.

### 6.10. Integration

Interdisciplinary research is significant during COVID-19 in order to implement it in smart communities. Many research areas, such as deep learning, machine learning, AI, privacy and security, computer networking, wireless networking, and predictive analysis, are necessary to integrate for collaborative research [89,90].

### 6.11. Privacy and Security 

One of the significant challenges of the deployment of the IoT in healthcare is still the privacy and security of the user data. If the challenges related to privacy and security are tackled successfully, standards of the IoT related to healthcare can be improved. The IoT operates in a heterogeneous network, and the primary responsibility of the IoT is the exchange of information between different devices and different internet connections. Therefore, it is challenging to assure the privacy and security of every device and connection. It is compulsory to ensure the user’s data privacy during IoT deployment during COVID-19. 

There are many aspects related to security and privacy, such as resource constraints (storage, processing, computation), physical security (tampering with medical devices), secure routing protocols, and data transparency (monitoring and management of patient data). For example, suppose that a COVID-19 patient went to the healthcare center for testing. The patient information is noted on the helpdesk, and then his information is uploaded to the server/database of the concerned healthcare center. The data are then transferred to the hospital’s private cloud, and from the hospital, the information is transferred to the concerned city administration to monitor patient activities. The data are transferred from many sensors, devices, and network connections and hence are very vulnerable. To protect the information of the user on a cloud-based IoT platform, a secure authentication mechanism along with a communication control and access control model are required.

Different IoT applications are important in every field of life. Table 1 shows smart applications and the challenges in the target industries that can affect IoT performance.

## 7. Conclusions

The IoT is considered the most promising technology for data transfer over interrelated networks without human interruption. The IoT offers services for the patient to share health-related data with a specific medical doctor and healthcare system in real time. However, many challenges exist in the IoT interrelated to the healthcare system that should be addressed to improve the healthcare system. 

This paper describes the role of the IoT in healthcare systems, especially during COVID-19. It describes the use of technology, architecture, elements, and application of the IoT relevant to healthcare systems. These applications can execute resourcefully and perform an important responsibility throughout COVID-19 and other diseases.

Similarly, a more innovative technology is explained and provides a stage for additional research and IoT healthcare system upgrading. Barriers are explained in detail, and upcoming recommendations will progress the healthcare system by eliminating the insufficiencies in IoT apps and technologies. By removing those barriers, a more innovative IoT healthcare system can be accomplished with more facilities and at minimum price to the healthcare system. This paper describes applications for future technology in the IoT along with current barriers for the healthcare system, COVID-19, and upcoming epidemics.

## Figures and Tables

**Table 1:** Challenges of the Target Industry.

**Target Industry**	**Functionalities**	**Challenges**
City	Traffic situation, pollution monitoring, parking, detection of virus and crime	QoS, mobility, SoP, performance evaluation, interpretability, E-management, policy and guidelines, implementation, integration, privacy and security
Building	Energy efficiency and maintenance, fire alarm, monitoring, etc.	SoP, performance evaluation, interpretability, policy and guidelines, implementation, integration, privacy and security
Home	TV, AC, lightning, doors, alarm, security cameras, etc.	Policy and guidelines, privacy and security
Grid	Power distribution and generation	Smartphone, QoS, mobility, performance evaluation, interpretability, integration
Industry	Robots, PLCs, conveyer built, etc.	Smartphone, QoS, mobility, performance evaluation, interpretability, integration
Agriculture	Robots, tractor, drones, etc.	Smartphone, QoS, mobility, performance evaluation, interpretability, E-management, policy and guidelines, implementation, integration, privacy and security
Education	Switches, routers, hubs, etc.	Mobility, SoP, E-management, policy and guidelines, privacy and security
Healthcare systems	Body sensors, patient monitoring drugs, tags, etc.	QoS, mobility, performance evaluation, interpretability, E-management, policy and guidelines, implementation, integration, privacy and security

## Data Availability

Not applicable.
